# A Segmentation Network with Two Distinct Attention Modules for the Segmentation of Multiple Renal Structures in Ultrasound Images

**DOI:** 10.3390/diagnostics15151978

**Published:** 2025-08-07

**Authors:** Youhe Zuo, Jing Li, Jing Tian

**Affiliations:** 1 Department of Ultrasound, Second Hospital of Tianjin Medical University, Tianjin 300211, China; youhezuo@tmu.edu.cn (Y.Z.); jing0928@tmu.edu.cn (J.L.); 2 Department of Urology, Second Hospital of Tianjin Medical University, Tianjin 300211, China

**Keywords:** renal structures, ultrasound images, encoder–decoder structure, attention mechanisms, multi-head self-attention mechanism

## Abstract

**Background/Objectives**: Ultrasound imaging is widely employed to assess kidney health and diagnose renal diseases. Accurate segmentation of renal structures in ultrasound images plays a critical role in the diagnosis and treatment of related kidney diseases. However, challenges such as speckle noise and low contrast still hinder precise segmentation. **Methods:** In this work, we propose an encoder–decoder architecture, named MAT-UNet, which incorporates two distinct attention mechanisms to enhance segmentation accuracy. Specifically, the multi-convolution pixel-wise attention module utilizes the pixel-wise attention to enable the network to focus more effectively on important features at each stage. Furthermore, the triple-branch multi-head self-attention mechanism leverages the different convolution layers to obtain diverse receptive fields, capture global contextual information, compensate for the local receptive field limitations of convolution operations, and boost the segmentation performance. We evaluate the segmentation performance of the proposed MAT-UNet using the Open Kidney US Data Set (OKUD). **Results:** For renal capsule segmentation, MAT-UNet achieves a Dice Similarity Coefficient (DSC) of 93.83%, a 95% Hausdorff Distance (HD95) of 32.02 mm, an Average Surface Distance (ASD) of 9.80 mm, and an Intersection over Union (IOU) of 88.74%. Additionally, MAT-UNet achieves a DSC of 84.34%, HD95 of 35.79 mm, ASD of 11.17 mm, and IOU of 74.26% for central echo complex segmentation; a DSC of 66.34%, HD95 of 82.54 mm, ASD of 19.52 mm, and IOU of 51.78% for renal medulla segmentation; and a DSC of 58.93%, HD95 of 107.02 mm, ASD of 21.69 mm, and IOU of 43.61% for renal cortex segmentation. **Conclusions:** The experimental results demonstrate that our proposed MAT-UNet achieves superior performance in multiple renal structure segmentation in ultrasound images.

## 1. Introduction

Ultrasound imaging is one of the most important techniques to assess kidney structures which is widely used in clinical practice. Owing to the safety and wide availability of ultrasound imaging, it is extensively employed to diagnose many renal diseases [1]. Ultrasound is a highly effective technique for assessing renal lesions [2]. Accurate identification and segmentation of the kidney structures in ultrasound images are essential for reliable diagnosis and effective treatment planning. Segmenting multiple renal structures in ultrasound images, such as the renal capsule, central echo complex, medulla, and cortex, can enhance clinical decision-making by enabling more objective assessment of kidney morphology, thereby reducing the potential for diagnostic errors and improving treatment planning. However, inherent limitations such as speckle noise, low contrast, and operator dependency hinder the precise delineation of anatomical boundaries. Moreover, traditional manual segmentation performed by clinicians is time-consuming and prone to variability. To address these challenges, automatic segmentation methods based on deep learning have emerged as promising solutions to improve diagnostic efficiency, enhance accuracy, and reduce time costs. In recent years, artificial intelligence (AI) has played a pivotal role in the development of medical image analysis. With the development of Convolutional Neural Networks (CNNs), the intelligence and automation of medical imaging analysis have been significantly enhanced. Inspired by the Fully Convolutional Network (FCN) [3], which utilizes convolution layers for pixel-wise image segmentation, U-Net [4] was proposed for medical image segmentation. U-Net adopts a characteristic U-shape network with an encoder–decoder structure with skip connections that effectively preserve feature information. Due to the effectiveness and simplicity of U-Net in medical image segmentation tasks, U-Net has become a widely used backbone in medical image segmentation, and many advanced variants based on this architecture have demonstrated remarkable performance. For the 3D medical image segmentation tasks, 3D U-Net [5] was designed based on the U-shape network architecture of the original 2D U-Net. He et al. [6] proposed ResNet to solve the difficulty of deeper network training, which introduces the residual blocks to mitigate the problem of the vanishing gradient. Hu et al. [7] developed an SENet which incorporates the Squeeze-and-Excitation (SE) block to focus on channel-wise feature information. In another similar attention-mechanism-related work, the Attention U-Net [8] integrates attention gates (AGs) into the U-Net to better focus on relevant features for medical image segmentation. For a common variant of U-Net, Zhou et al. [9] redesigned the skip pathways based on U-Net to reduce the semantic divergence between the encoder and decoder feature maps. Chen et al. [10] presented a DeepLabv3+ model, which utilizes the atrous convolution layer, an Xception backbone, and atrous spatial pyramid pooling to enhance feature extraction. To address the problem of spatial information loss, Gu et al. [11] designed a context encoder network (CE-Net) for 2D medical image segmentation. CE-Net integrates the context extractor to obtain high-level feature information and preserve spatial information. Inspired by the success of Vision Transformer (ViT) [12] in computer vision tasks, Chen et al. [13] presented a TransUNet, which combines the Transformer and UNet architecture to capture the long-range dependencies. Similarly, Cao et al. [14] proposed SwinUNet based on the U-shaped architecture and Swin Transformer [15] for medical image segmentation. Zhu et al. [16] presented a DBUNet, which leverages a Deep Feature Aggregation Module (DFAM) and a Shallow Feature Optimization Module (SFOM) to enhance segmentation performance in ultrasound images.

With the rapid development of CNNs and Transformers in recent years, numerous deep learning methods have been proposed for renal structure segmentation in ultrasound images. Wu et al. [17] designed a cascaded FC-DenseNet that consists of a coarse segmentation model and a fine segmentation model for kidney segmentation. Considering the importance of kidney structure boundary information, Yin et al. [18] proposed a boundary distance regression network. Chen et al. [19] developed a multi-scale and deep-supervised encoder–decoder network that utilizes a pyramid pooling module for kidney structure segmentation in ultrasound images. Chen et al. [20] introduced an MBANet that integrates a multi-scale feature pyramid (MSFP) and multi-branch encoder (MBE). In the MBANet, the MSFP is used to enhance the network’s ability to obtain details at different scales, while the MBE is used to reduce the information loss and improve the segmentation performance. Additionally, a multi-scale fusion block (MFBlock) is embedded within the MBE to obtain multi-scale feature information. Valente et al. [21] conducted experiments to compare the segmentation performance of seven existing segmentation networks across multiple renal structures in ultrasound images. To further enhance kidney segmentation performance, Chen et al. [22] proposed an encoder–decoder network with a hybrid attention mechanism. Wang et al. [23] developed a Short-Term Dense Concatenate network (STDC) for kidney segmentation in dynamic ultrasound images. Chen et al. [24] designed an MBDSNet with a multi-branch and deep supervision network for kidney structure segmentation, and used the kidney boundary detection module to guide the network to effectively segment the kidney with complete contour. Chang et al. [25] presented a W-Net based on nnU-Net [26] and trained two stages for multi-center kidney segmentation. For the kidney ultrasound image segmentation, Khan et al. [27] proposed an MLAU-Net incorporating deep supervision and an attention gate to enhance segmentation performance. Despite these advancements, accurately segmenting internal renal structures in ultrasound images remains a significant challenge due to the complex and variable nature of internal anatomical features. In this paper, our contributions can be summarized as follows:

(1) We explore deep-learning-based methods for the segmentation of multiple renal structures in ultrasound images and propose a novel segmentation model named MAT-UNet, which demonstrates high reliability, accuracy, and robustness.
(2) We design a multi-convolution pixel-wise attention module (MCPAM), which utilizes convolution layers of different kernel sizes and pixel-wise attention to lead the network to focus on more important features.
(3) To enhance the model’s ability to capture features, we develop a triple-branch multi-head self-attention mechanism (TBMSM) at the bottom of MAT-UNet. The triple-branch multi-head self-attention mechanism uses three convolution layers with different kernel sizes to obtain different receptive fields and learn the global contextual features, and employs three multi-head self-attention mechanisms to effectively learn global contextual information.

## 2. Materials and Methods

### 2.1. Network Architecture

#### 2.1.1. Overall

**Figure 1:**
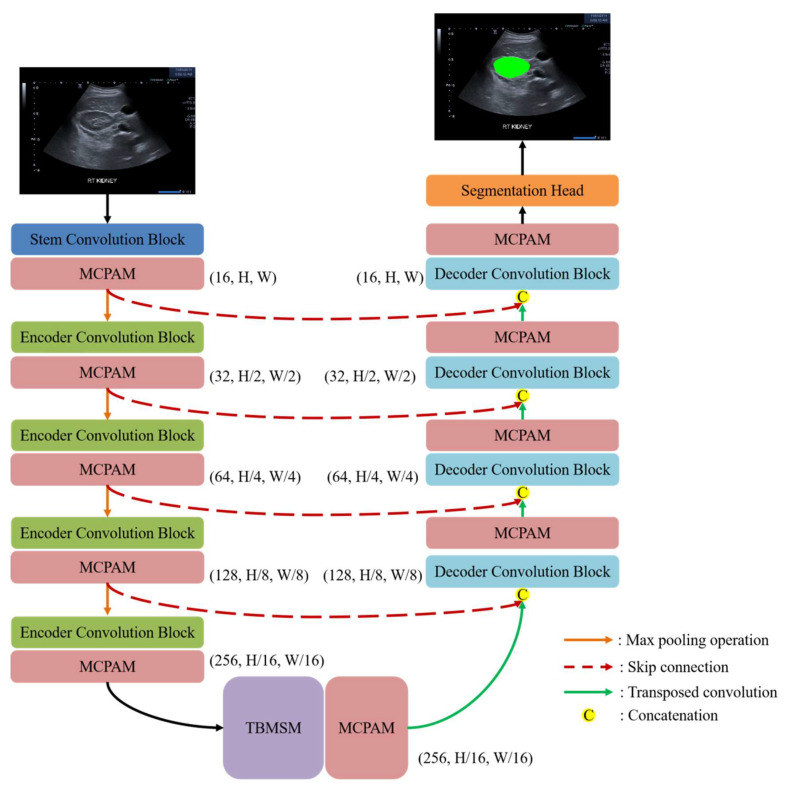
The overall network architecture of the proposed MAT-UNet. MCPAM and TBMSM refer to the multi-convolution pixel-wise attention module and the triple-branch multi-head self-attention mechanism that we proposed.

As shown in Figure 1, the overall architecture of MAT-UNet comprises three main components: the encoder part, the triple-branch multi-head self-attention mechanism, and the decoder part. Between the encoder and decoder, four skip connections are employed to transfer the feature maps from the encoder stages to the corresponding decoder stages for preserving spatial information. Following the decoder, a segmentation head block is used to generate the final segmentation results. The segmentation head block comprises a convolution layer with a kernel size of 1 × 1 and a softmax activation layer.

#### 2.1.2. Encoder

**Figure 2:**
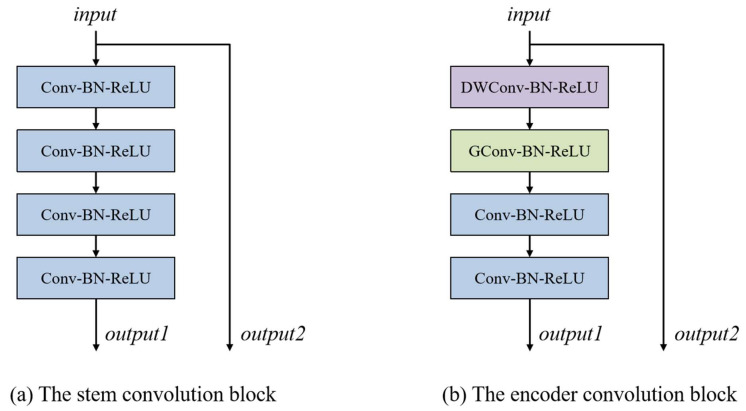
(**a**) and (**b**) illustrate the structures of the stem convolution block and encoder convolution block, respectively. The Conv in the stem convolution block and encoder convolution block stands for a convolution layer with a kernel size of 3 × 3. The DWConv and GConv in the encoder convolution block represent a depth-wise convolutional layer and a group convolutional layer, respectively, both with a kernel size of 3 × 3. The BN and ReLU represent a Batch Normalization layer and a ReLU activation layer, respectively.

As Figure 2 shows, two distinct convolution blocks are employed in the encoder. At the top stage of the encoder branch, a stem convolution block is used to extract features from the input image. As illustrated in Figure 2a, the stem convolution block consists of four convolution layers with a kernel size of 3 × 3, each followed by a Batch Normalization layer and a ReLU activation layer. The stem convolution block increases the number of channels from 1 to 16. Following the top stage, four encoder convolution blocks are used in the subsequent stages of the encoder. The structure of the encoder convolution block is shown in Figure 2b. Each encoder convolution block employs a depth-wise convolution layer and a group convolution layer, both with a 3 × 3 kernel size. After that, two common convolution layers with a 3 × 3 kernel size are used to further enhance feature extraction capability. The operation of an encoder convolution block can be formulated as follows:

(1) $$O_{1}=\sigma (BN(DWConv(I)))$$

(2) $$O_{2}=\sigma (BN(GConv(O_{1})))$$

(3) $$O_{3}=\sigma (BN(Conv(\sigma (BN(Conv(O_{2}))))))$$

where I, $O_{1}$, $O_{2}$, and $O_{3}$ represent the input and output feature maps of the operations, respectively. σ(.) denotes the ReLU activation layer and *BN*(.) represents the Batch Normalization layer. DWConv(.), GConv(.), and Conv(.) stand for a depth-wise convolution layer, a group convolution layer, and a common convolution layer, respectively. After each encoder convolution block, the number of feature map channels doubles. The proposed multi-convolution pixel-wise attention module is used at the end of each encoder stage to guide the encoder in learning more useful feature information. A max pooling operation with a 2 × 2 stride is used after each encoder stage to reduce the size of feature maps. After the max pooling operation, the size of feature maps is reduced by half.

#### 2.1.3. Decoder

**Figure 3:**
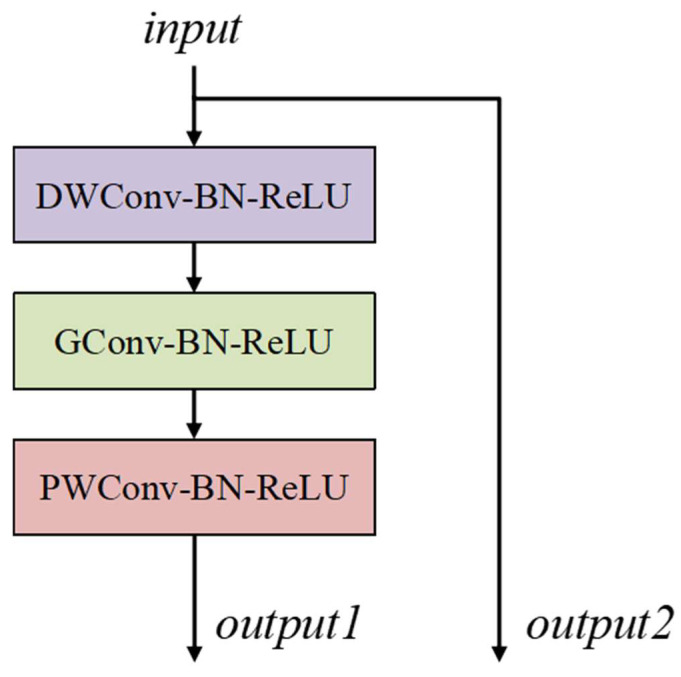
The structure of the decoder convolution block. DWConv, GConv, and PWConv represent the depth-wise convolution layer with a 3 × 3 kernel size, group convolution layer with a 3 × 3 kernel size, and point-wise convolution layer, respectively. BN and ReLU denote the Batch Normalization layer and ReLU activation layer, respectively.

In the decoder part, a transposed convolution layer with a 2 × 2 kernel size and a 2 × 2 stride is used to double the size while halving the number of feature map channels. These feature maps are concatenated with other feature maps from the encoder via skip connections. To make the decoder more lightweight, we design the decoder convolution block as illustrated in Figure 3. Each decoder convolution block consists of a depth-wise convolution layer with a kernel size of 3 × 3, a group convolution layer with a kernel size of 3 × 3, and a point-wise convolution layer with a 1 × 1 kernel size. A Batch Normalization layer and a ReLU layer are used after each convolution layer. The decoder convolution block can be formulated as follows:

(4) $$D_{1}=\sigma (BN(DWConv(I)))$$

(5) $$D_{2}=\sigma (BN(GConv(D_{1})))$$

(6) $$D_{3}=\sigma (BN(PWConv(D_{2})))$$

where I, $D_{1}$, $D_{2}$, and $D_{3}$ represent the input feature maps and the outputs of the corresponding operations, respectively. *BN*(.) and σ(.) represent the Batch Normalization layer and ReLU activation layer, respectively. DWConv(.), GConv(.), and PWConv(.) represent the depth-wise convolution layer, group convolution layer, and point-wise convolution layer, respectively. At the end of each decoder stage, the proposed multi-convolution pixel-wise attention module is employed to learn more important information from the feature maps.

#### 2.1.4. Multi-Convolution Pixel-Wise Attention Module

**Figure 4:**
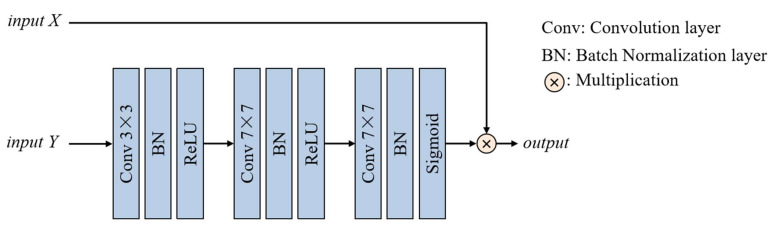
The structure of the multi-convolution pixel-wise attention module.

To enhance the model’s ability to focus on important features, we introduce the multi-convolution pixel-wise attention module (MCPAM) that reweights feature representations at a fine-grained level. This module generates pixel-wise attention weights based on the input features of the stage, effectively amplifying responses that are highly relevant to the target structures. The multi-convolution pixel-wise attention module addresses the limitations of traditional channel-level attention mechanisms in spatial granularity, and achieves more refined feature selection and response enhancement through pixel-wise weighting operations, especially when dealing with complex anatomical structures. The structure of the multi-convolution pixel-wise attention module is shown in Figure 4, which is used to obtain more informative and task-relevant features. Specifically, the module takes two inputs, *X* and *Y*, which provide different useful information from the feature maps. *Y* is input through a convolution layer with a 3 × 3 kernel size, a Batch Normalization layer, and a ReLU activation layer, and then the channels of input *Y* become the same as input *X*. Then, two 7 × 7 kernel size convolution layers are utilized to obtain larger receptive fields. Finally, a Sigmoid activation layer is used to obtain feature map information weights, and to multiply them with the input *X* directly. The operations of the MCPAM can be formally defined as follows:

(7) $$Y_{1}=\sigma (BN({Conv}_{3\times 3}(Y)))$$

(8) $$Y_{2}=\sigma (BN({Conv}_{7\times 7}(Y_{1})))$$

(9) $$Y_{3}=\delta (BN({Conv}_{7\times 7}(Y_{2})))\times X$$

where *Y* and *X* are the input *Y* and input *X*, respectively. ${Conv}_{3\times 3}(.)$ and ${Conv}_{7\times 7}(.)$ represent a convolution layer with a kernel size of 3 × 3 and 7 × 7, respectively. *BN*(.), σ(.), and δ(.) represent the Batch Normalization layer, ReLU activation layer, and Sigmoid activation layer, respectively.

#### 2.1.5. Triple-Branch Multi-Head Self-Attention Mechanism

**Figure 5:**
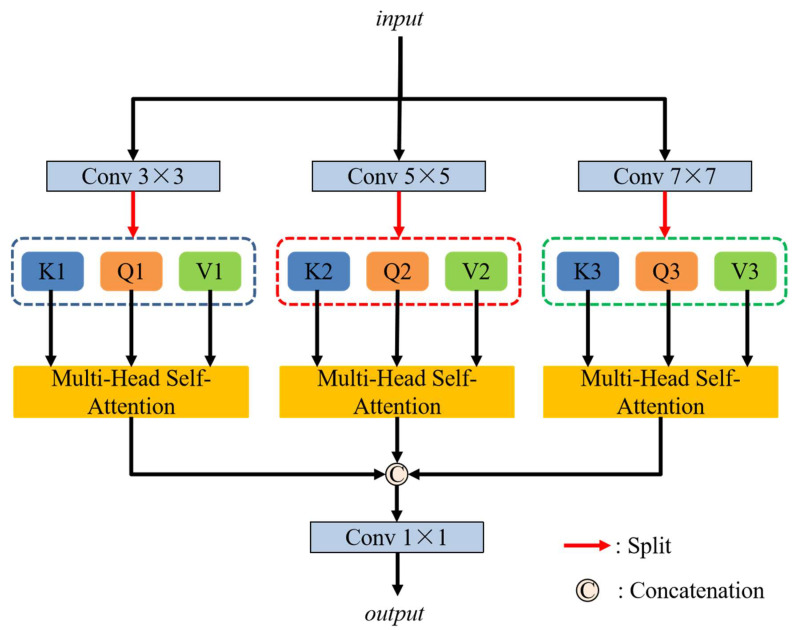
The structure of the triple-branch multi-head self-attention mechanism (TBMSM).

Ultrasound images often suffer from low resolution, speckle noise, and indistinct anatomical boundaries, which cause substantial challenges to accurate segmentation. The proposed triple-branch multi-head self-attention mechanism (TBMSM) is designed to enhance the model’s ability to capture multi-scale contextual information in complex ultrasound images. By incorporating triple multi-head self-attention branches with diverse receptive fields, the module enables the model to adaptively capture spatial dependencies across varying scales. The multi-head self-attention mechanism facilitates long-range feature interaction, which is essential for learning the global context features of anatomical targets. As Figure 5 illustrates, the triple-branch multi-head self-attention mechanism is proposed for capturing multi-scale contextual information and enhancing the segmentation performance. Three different kernel size convolution layers are used in three branches, respectively, to obtain different receptive fields. After these three convolution layers, three different feature maps are obtained, and the channel dimension is increased threefold. Then, each of these feature maps is evenly divided into three parts to generate the queries (*Q*), keys (*K*), and values (*V*). These operations can be calculated as follows:

(10) $$Q_{1},K_{1},V_{1}=Split({Conv}_{3\times 3}(E))$$

(11) $$Q_{2},K_{2},V_{2}=Split({Conv}_{5\times 5}(E))$$

(12) $$Q_{3},K_{3},V_{3}=Split({Conv}_{7\times 7}(E)$$

where E is the input of the triple-branch multi-head self-attention mechanism. ${Conv}_{k\times k},\;k\in \{3,\;5,\;7\}$ represents the different convolution layer with kernel sizes of *k* × *k*, k∈{3, 5, 7}. $Q_{i},K_{i},V_{i},\;i\in \{1,\;2,\;3\}$ are the queries (*Q*), keys (*K*), and values (*V*) of different branches.

Then $Q_{i},K_{i},V_{i},\;i\in \{1,\;2,\;3\}$ are respectively input the multi-head self-attention module:

(13) $$Att(Q,K,V)=Softmax(\frac{QK^{Τ}}{\sqrt{d}})V$$

where Att(.) means the multi-head self-attention operation and *d* represents three times the number of channels in the input image.

The outputs from three branches are concatenated for fusing attention information, and then a 1 × 1 kernel size convolution layer is used to reduce the number of output channels. These operations can be defined as follows:

(14) $$N=Concat(Att(Q_{1},K_{1},V_{1}),Att(Q_{2},K_{2},V_{2}),Att(Q_{3},K_{3},V_{3}))$$

(15) $$M={Conv}_{1\times 1}(N)$$

where Concat(.) and ${Conv}_{1\times 1}(.)$ represent the concatenation operation and a convolution layer with a 1 × 1 kernel size. $Q_{i},K_{i},V_{i},\;i\in \{1,\;2,\;3\}$ are the queries (*Q*), keys (*K*), and values (*V*) from three different branches. *M* is the output of the triple-branch multi-head self-attention mechanism.

### 2.2. Dataset

In our experiments, we utilize the Open Kidney US Data Set (OKUD) [28] which comprises 534 ultrasound images along with their corresponding labels. The total of 534 B-mode ultrasound images consists of 514 unique images, along with 20 additional duplicate copies originating from the 514 images. The B-mode ultrasound images were acquired between January 2015 and September 2019 from patients undergoing kidney ultrasound scans due to clinical indications. The dataset covers a diverse range of ultrasound vendors, including SonoSite, Acuson, General Electric (GE), Toshiba, Siemens, and Philips. The dataset provides two different label sets annotated independently by two experts. To ensure consistency and reliability in evaluation, we adopt the label files annotated by the second expert. The label files include two subfolders: one subfolder includes the labels for the renal capsule, and another subfolder includes the labels for the central echo complex, renal medulla, and renal cortex. This division of label files facilitates the segmentation of both the outer and internal renal structures, enabling a comprehensive assessment of the model’s performance across different anatomical regions.

Following the label file division of the dataset, we conduct two separate segmentation tasks for this dataset. The first task focuses on segmenting the renal capsule. Subjects lacking labels for the renal capsule are excluded, resulting in a total of 481 subjects with valid labels. The 481 subjects are randomly split into 341 subjects for training, 49 subjects for validation, and 97 subjects for testing. The second task is to segment multiple internal renal structures including the central echo complex, renal medulla, and renal cortex. A subject is excluded if any of the three anatomical regions are missing in the labels. After this filtering process, 323 subjects remain and are randomly divided into 226 subjects for training, 32 subjects for validation, and 65 subjects for testing. These two separate tasks enable the evaluation of both single-structure and multi-structure segmentation performance, providing insights into the model’s generalization across different levels of anatomical complexity.

### 2.3. Implementation Details

For each task, the batch size is set to 4 during the training phase, and the model is trained for 30,000 iterations. The stochastic gradient descent (SGD) with a momentum of 0.9 and weight decay of 0.0001 is used as the optimizer for model training. The initial learning rate is set to 0.01, and a polynomial learning rate decay strategy is used to reduce the learning rate. The decay strategy can be formulated as follows:

(16) $$lr={lr}_{i}\times {\left(1-\frac{T_{c}}{T_{m}}\right)}^{0.9}$$

where lr, ${lr}_{i}$, $T_{c}$, and $T_{m}$ represent the current learning rate, initial learning rate, current training iteration, and maximum number of training iterations, respectively. The patch size of input images is cropped to 512 × 512, and their intensity values are normalized to the range [0, 1]. The data augmentation methods including rotation, flipping, Gaussian noise, and color jitter are used in the model training phase to improve the diversity of the dataset. The model achieving the highest DSC score on the validation set is used for testing. All experiments are conducted in Ubuntu 20.04 with an NVIDIA RTX 3080 Ti GPU. The model is implemented using Python 3.10.16 and PyTorch framework 2.1.1.

### 2.4. Loss Function

For more robust segmentation performance, a combined loss function of Dice loss and Cross-Entropy loss is used for model training. The combined loss function is commonly used in various medical image segmentation tasks and focuses on pixel-level accuracy and ground truth regions, which can help handle class imbalance. The total loss functions are defined as follows:

(17) $${Loss}_{t}=\lambda \times \left({Loss}_{d}+{Loss}_{c}\right)$$

where ${Loss}_{t}$, ${Loss}_{d}$, and ${Loss}_{c}$ stand for the total loss function, Dice loss, and Cross-Entropy loss, respectively. λ is set to 0.5 to balance the two different loss functions.

### 2.5. Metrics

Four evaluation metrics are utilized to assess the quality of segmentation results: Dice Similarity Coefficient (DSC), 95% Hausdorff Distance (HD95), Average Surface Distance (ASD), and Intersection over Union (IOU). Higher DSC and IOU values indicate better segmentation performance, while lower values of the HD95 and ASD indicate better boundary alignment. HD95 is a variant of HD that uses the 95th percentile of distances instead of the maximum, making it less sensitive to outliers. These metrics are defined as follows:

(18) $$DSC=\frac{2\times |A\cap B|}{\left|A|+|B\right|}$$

(19) $$HD95=0.95\times max\{\underset{a\in A}{\max}\left(d\left(a,B\right)\right),\underset{b\in B}{\max}\left(d\left(b,A\right)\right)\}$$

(20) $$ASD=\frac{1}{S(A)+S(B)}\times \{\sum_{b\in S\left(B\right)}d\left(s_{b},S\left(A\right)\right)+\sum_{a\in S\left(A\right)}d\left(s_{a},S\left(B\right)\right)\}$$

(21) $$IOU=\frac{\left|A\cap B\right|}{\left|A\cup B\right|}$$

where *A* and *B* represent the ground truth and segmentation result, respectively. d(·) represents the shortest Euclidean Distance. S(·) represents the set of surface pixels of the image.

## 3. Results

### 3.1. Comparison Results of Renal Capsule

**Figure 6:**
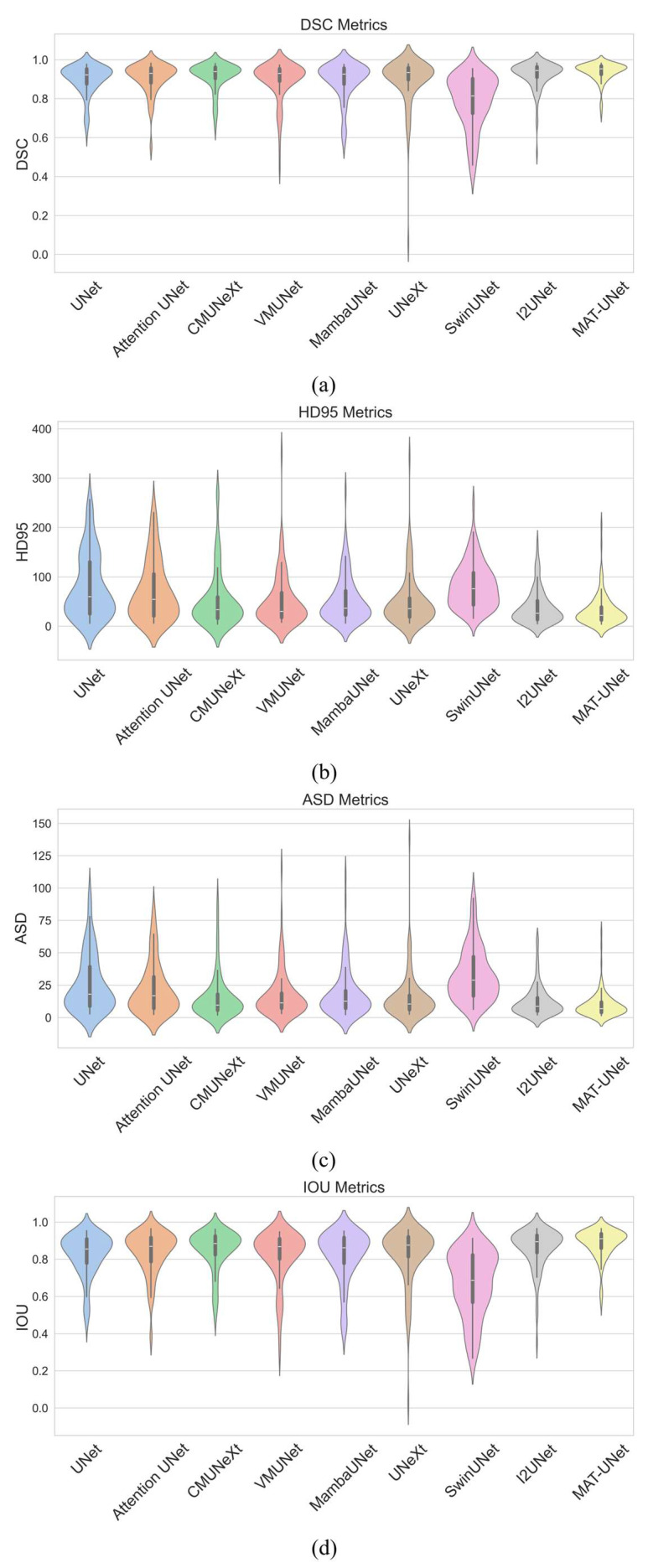
The violin plots of the comparison results. (**a**), (**b**), (**c**), and (**d**) are the plots of DSC, HD95, ASD, and IOU metrics, respectively.

**Figure 7:**
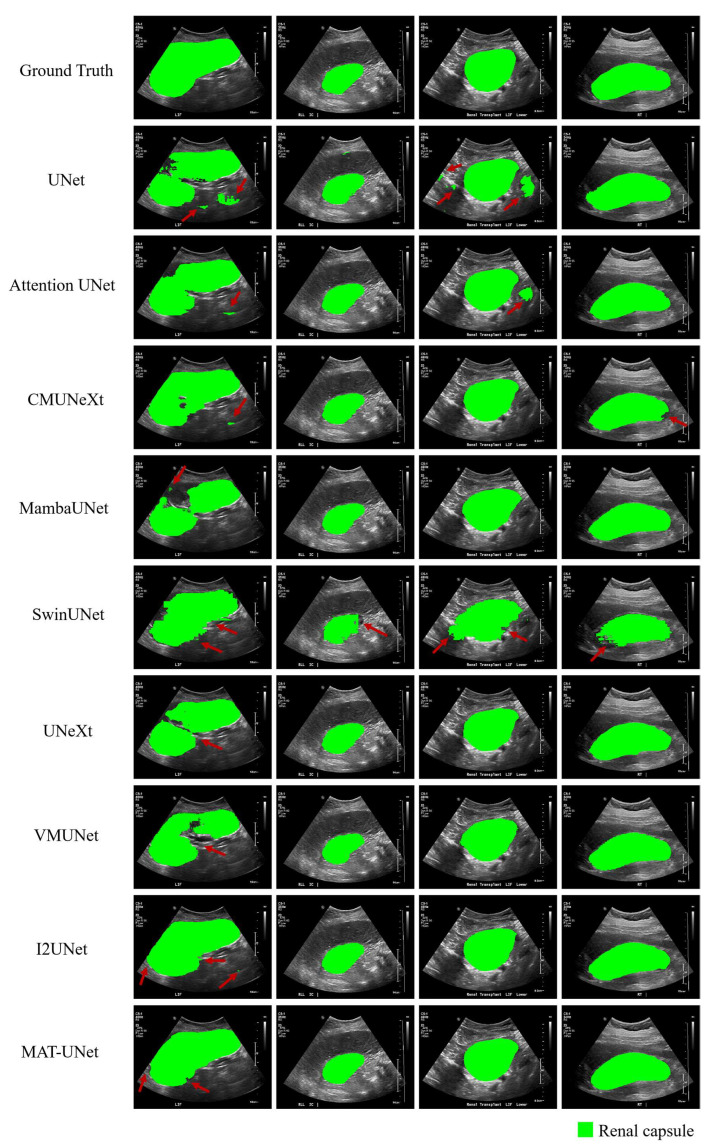
The visualization of segmentation results from comparison methods. The green region represents the renal capsule structure.

To evaluate the effectiveness of the proposed model, we conducted comparative experiments under consistent experimental settings, in which both our model and the baseline models were trained from scratch without any pre-training. We compare our model with several state-of-the-art (SOTA) segmentation models including UNet [4], Attention UNet [8], CMUNeXt [29], VMUNet [30], MambaUNet [31], UNeXt [32], SwinUNet [14], and I2UNet [33]. The comparison results are listed in Table 1. As Table 1 shows, our method achieves a DSC of 93.83%, HD95 of 32.02 mm, ASD of 9.80 mm, and IOU of 88.74% for renal capsule segmentation. Our model outperforms all other compared methods across all four evaluation metrics. The I2UNet achieves the second-best results in four evaluation metrics. Compared to I2UNet, MAT-UNet improves the DSC and IOU by 1.69% and 2.62%, respectively, while reducing the HD95 and ASD by 6.00 mm and 2.57 mm, respectively. The violin plots in Figure 6 further illustrate the segmentation performance distribution of each model. According to Figure 6, MAT-UNet demonstrates superior stability and accuracy in terms of DSC and IOU metrics, whereas UNetXt and SwinUNet exhibit relatively poor performance on some samples. For the HD95 and ASD metrics, our proposed approach also achieves competitive performance and robust consistency. The segmentation results of comparison methods are presented in Figure 7, where MAT-UNet provides clearer and more accurate delineation of anatomical structures than the other models. In the first case, except the I2UNet and MAT-UNet, none of the other models are able to effectively segment the complete renal capsule region. In the second case, SwinUNet struggles to produce a relatively complete segmentation of the renal capsule. In the third case, UNet and Attention UNet incorrectly segment the isolated areas outside the renal capsule. In the fourth case, both CMUNeXt and SwinUNet exhibit inferior segmentation quality compared to the remaining models. Overall, the comparison results and visualizations of the renal capsule demonstrate that MAT-UNet achieves superior performance for renal capsule segmentation in ultrasound images.

### 3.2. Comparison Results of Internal Renal Structures

**Figure 8:**
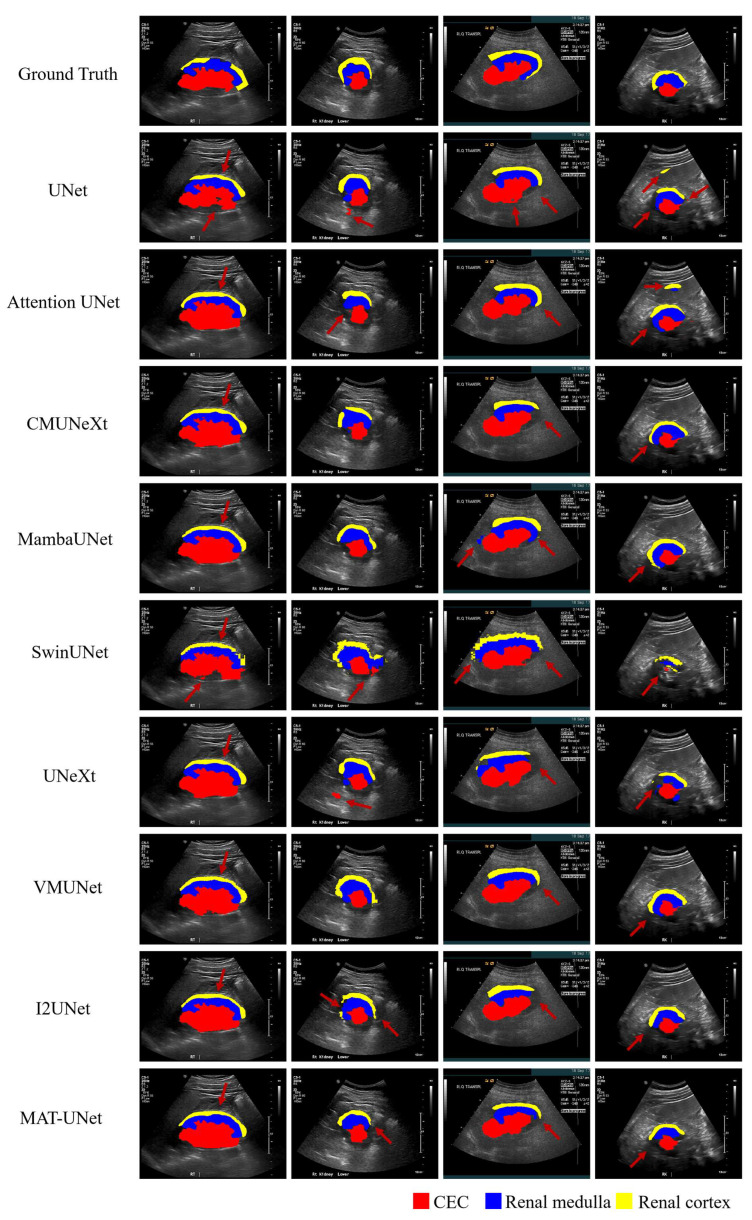
The visualization of segmentation results from comparison methods. The red, blue, and yellow regions represent the central echo complex (CEC), renal medulla, and renal cortex structures, respectively.

For task 2, we also compare our method with UNet, Attention UNet, CMUNeXt, VMUNet, MambaUNet, UNeXt, SwinUNet, and I2UNet in the same experimental conditions and settings. The comparison results are presented in Table 2. According to Table 2, for the central echo complex (CEC), our method achieves a DSC of 84.34%, HD95 of 35.79 mm, ASD of 11.17 mm, and IOU of 74.26%. Among these metrics, the DSC and IOU values that our method achieves are the highest compared to the other methods. For the renal medulla, our method achieves a DSC of 66.34%, HD95 of 82.54 mm, ASD of 19.52 mm, and IOU of 51.78%. The DSC, ASD, and IOU metrics of the renal medulla that our method achieves are the best among all methods. For the renal cortex, our method achieves a DSC of 58.93%, HD95 of 107.02 mm, ASD of 21.69 mm, and IOU of 43.61%. The DSC, ASD, and IOU metrics that our method achieves are the best values compared to the other methods. The visualization of our model and the compared models is shown in Figure 8. As depicted in Figure 8, the renal regions segmented by our method exhibit fewer errors compared to other methods. In the first case, all methods incorrectly classify part of the renal medulla as the renal cortex. In the remaining three cases, our method demonstrates superior segmentation performance.

### 3.3. Ablation Results

To evaluate the effectiveness of the proposed components, we conduct a series of ablation experiments. We regard the network architecture of MAT-UNet without the multi-convolution pixel-wise attention module and triple-branch multi-head self-attention mechanism as the baseline. We use the baseline for the first ablation experiment, the baseline with the multi-convolution pixel-wise attention module for the second ablation experiment, the baseline with the triple-branch multi-head self-attention mechanism for the third ablation experiment, and the baseline with the multi-convolution pixel-wise attention module and the triple-branch multi-head self-attention mechanism for the fourth ablation experiment. The results of the ablation experiments are listed in Table 3. According to the ablation results, the baseline equipped with the multi-convolution pixel-wise attention module and the baseline with the triple-branch multi-head self-attention mechanism both improve segmentation performance compared to the baseline. Especially, the baseline with the triple-branch multi-head self-attention mechanism improves the segmentation performance significantly. In addition, compared to the baseline, the proposed MAT-UNet improves DSC by 2.59% and IOU by 4.03%, reducing HD95 by 29.61 mm and ASD by 10.05 mm. The ablation experiments demonstrate the effectiveness of the proposed modules for renal structure segmentation in ultrasound images.

## 4. Discussion

Accurate segmentation of the renal structures in ultrasound images holds significant clinical importance. However, the inherent complexity and variability of renal structures present substantial challenges for precise segmentation. To mitigate these challenges, we propose a U-shaped encoder–decoder network architecture named MAT-UNet. Aiming to enhance feature extraction capacity, we redesign the convolution operations in both the encoder and decoder, and introduce the depth-wise, group, and point-wise convolution layers. The MAT-UNet integrates the proposed multi-convolution pixel-wise attention module (MCPAM) and triple-branch multi-head self-attention mechanism (TBMSM). The multi-convolution pixel-wise attention module utilizes three convolution layers to obtain the pixel-wise attention weights, and guides the model to focus on more important feature information. The triple-branch multi-head self-attention mechanism introduces three parallel branches, each using different kernel size convolution layers for three different multi-head self-attention blocks to capture diverse contextual dependencies.

To validate the effectiveness of the proposed method for kidney structure segmentation in ultrasound images, we compare MAT-UNet with several state-of-the-art (SOTA) segmentation models. The comparative experiment results demonstrate that our method achieves superior performance in renal capsule segmentation. For the other four internal renal structure segmentations, our method achieves the best results in a total of eight metrics compared to the other models. Furthermore, to prove the contributions of the proposed multi-convolution pixel-wise attention module and triple-branch multi-head self-attention mechanism in the model, we conduct a series of ablation experiments. The ablation experiment results indicate that the proposed multi-convolution pixel-wise attention module and triple-branch multi-head self-attention mechanism both have positive effects for the model in kidney structure ultrasound image segmentation.

Although our proposed approach achieves excellent performance for multiple renal structure segmentation in ultrasound images, several problems remain to be discussed. First, compared to other comparison methods, the boundary evaluation indicators like HD95 and ASD which MAT-UNet obtains need further improvement. Second, due to the inherent variability and complex appearance of internal renal structures, the segmentation performance is not good enough, and further enhancement of the internal renal structure segmentation performance is still necessary. Therefore, improving the model’s sensitivity to intra-organ texture and subtle structural differences is essential for advancing its clinical applicability. Future work may focus on integrating advanced boundary refinement strategies or hybrid attention mechanisms to improve edge precision. Additionally, domain knowledge or anatomical priors can be incorporated to help the model better differentiate internal renal components, thereby boosting segmentation accuracy and robustness.

## 5. Conclusions

In this paper, we propose a novel segmentation network named MAT-UNet for the multiple renal structure segmentation in ultrasound images. The MAT-UNet is composed of a multi-convolution pixel-wise attention module (MCPAM) and a triple-branch multi-head self-attention mechanism (TBMSM) to enhance feature extraction capabilities. The results of comparative and ablation experiments demonstrate the superior segmentation performance, robustness, and effectiveness of the proposed MAT-UNet. In future work, we will continue our research on kidney ultrasound imaging and explore the application of other deep learning methods for the analysis of renal structures. Furthermore, we plan to conduct experiments on multi-center datasets in future studies to better demonstrate the generalization ability of our method.

## Figures and Tables

**Table 1:** The results of our method compared to other segmentation methods in terms of DSC, HD95, ASD, and IOU for the renal capsule segmentation task. The bold denotes the best value compared to others. * denotes a statistically significant difference (*p* < 0.05) based on the paired *t*-test comparing the proposed method with the other methods.

Methods	DSC (%)	HD95 (mm)	ASD (mm)	IOU (%)
UNet	89.95 *	81.02 *	25.80 *	82.54 *
Attention UNet	90.69 *	71.13 *	21.83 *	83.79 *
CMUNeXt	91.58 *	52.96 *	15.54 *	85.17 *
VMUNet	89.34 *	51.27 *	17.47 *	81.96 *
MambaUNet	89.33 *	53.31 *	18.41 *	81.89 *
UNeXt	89.50 *	50.39 *	15.48 *	82.60 *
SwinUNet	78.94 *	82.09 *	33.09 *	67.16 *
I2UNet	92.14 *	38.02 *	12.37 *	86.12 *
Ours	**93.83**	**32.02**	**9.80**	**88.74**

**Table 2:** The comparison results of the central echo complex, renal medulla, and renal cortex. CEC stands for central echo complex. The bold denotes the best value compared to others. * denotes a statistically significant difference (*p* < 0.05) based on the paired *t*-test comparing the proposed method with the other methods.

Structures	Methods	DSC (%)	HD95 (mm)	ASD (mm)	IOU (%)
CEC	UNet	81.48 *	50.10 *	16.45 *	70.40 *
	Attention UNet	81.42 *	55.43 *	16.68 *	70.34 *
	CMUNeXt	83.13	42.11	12.63	72.30
	VMUNet	80.73 *	42.70	12.35	69.63 *
	MambaUNet	79.62 *	46.15 *	13.68	68.48 *
	UNeXt	79.29 *	60.43 *	16.34 *	67.95 *
	SwinUNet	72.72 *	79.60 *	28.55 *	59.96 *
	I2UNet	82.70	**33.73**	**10.92**	72.86
	Ours	**84.34**	35.79	11.17	**74.26**
renal medulla	UNet	64.81	85.10	24.62 *	50.28
	Attention UNet	62.91 *	82.92	22.97 *	48.13 *
	CMUNeXt	63.71	88.91	27.71 *	48.71
	VMUNet	62.38 *	86.16	27.23 *	47.78 *
	MambaUNet	62.58	83.90	25.56 *	47.77 *
	UNeXt	60.39 *	87.36	24.67 *	45.76 *
	SwinUNet	52.41 *	112.59 *	42.89 *	37.74 *
	I2UNet	65.56	**75.48**	22.40	50.91
	Ours	**66.34**	82.54	**19.52**	**51.78**
renal cortex	UNet	57.04	112.92	26.82	41.53
	Attention UNet	58.30	120.28	27.51 *	42.62
	CMUNeXt	56.53	111.62	31.53 *	41.01
	VMUNet	53.40 *	114.95	35.81 *	38.36 *
	MambaUNet	54.40 *	119.38	33.35 *	39.14 *
	UNeXt	51.55 *	117.81	31.79 *	36.88 *
	SwinUNet	43.54 *	138.05 *	46.60 *	29.55 *
	I2UNet	57.35	**100.31**	25.74	42.00
	Ours	**58.93**	107.02	**21.69**	**43.61**

**Table 3:** The results of ablation experiments for the renal capsule segmentation task. MCPAM and TBMSM refer to the multi-convolution pixel-wise attention module and triple-branch multi-head self-attention mechanism, respectively. The bold denotes the best value compared to others. * denotes a statistically significant difference (*p* < 0.05) based on the paired *t*-test comparing the proposed method with the other methods.

Methods	DSC (%)	HD95 (mm)	ASD (mm)	IOU (%)
Baseline	91.24 *	61.63 *	19.85 *	84.71 *
Baseline + MCPAM	92.56 *	35.25	11.31	86.84 *
Baseline + TBMSM	93.26	37.06	10.49	87.81
Baseline + MCPAM + TBMSM	**93.83**	**32.02**	**9.80**	**88.74**

## Data Availability

The dataset used in this study is available upon request at https://github.com/rsingla92/kidneyUS (requested on 12 March 2025; received on 13 March 2025).
